# Caring for older men and women: whose caregivers are more distressed? A population-based retrospective cohort study

**DOI:** 10.1186/s12877-022-03583-6

**Published:** 2022-11-22

**Authors:** Wenshan Li, Douglas G. Manuel, Sarina R. Isenberg, Peter Tanuseputro

**Affiliations:** 1 School of Epidemiology and Public Health, University of Ottawa, Ottawa, ON Canada; 2 Bruyère Research Institute, Ottawa, ON Canada; 3 ICES uOttawa, Ottawa, ON Canada; 4 Clinical Epidemiology Program, Ottawa Hospital Research Institute, Ottawa, ON Canada; 5 Division of Palliative Care, Department of Medicine, University of Ottawa, Ottawa, Canada

**Keywords:** Caregiver distress, Care-receiver’s gender, Older men, Older women

## Abstract

**Background:**

To our knowledge, no population-based studies have examined whether family or friend caregivers of men and women differ in their experience of distress over time. Thus, we aimed to describe, on a population-level and longitudinally, how older men and women care-receivers differed in their health and care needs, compare their caregivers’ distress trajectories, and identify factors that contribute to the observed differences.

**Methods:**

This is a population-based, retrospective cohort study using routinely collected data. We examined longitudinally 485,407 community-dwelling Ontario residents, aged over 50 years, who have received at least one Residential Assessment Instrument-Home Care (RAI-HC) assessment between April 2008 and June 2015. Descriptive analyses were performed on the demographic characteristics, health profiles, and care needs of men and women. We also compared their caregivers’ baseline and one-year change in distress status. Logistic regressions were performed to examine if the effect of gender on caregiver distress is reduced after controlling for care-receiver’s health and functional status as well as their caregiver’s kinship and co-residence status.

**Results:**

Men (39.5% of our cohort) were frailer, required more care, were mostly cared for by their spouses (52%), and mostly lived with their caregiver (66%). In contrast, women (60.5%) were more likely cared for by their child/child-in-law (60%), less likely to live with caregivers (47%), and received less care. Caregivers of men were more likely to be distressed at baseline (27.7% versus 20.4% of women caregivers) and remain distressed (74.6% versus 69.5%) or become distressed (19.3% versus 14.3%) throughout the year. In logistic regression modelling, the effect of care-receiver’s gender on caregiver distress is reduced from an unadjusted odds ratio of 1.49 (95% CI: 1.47–1.51) to 1.17 (95% CI: 1.15–1.19) when care-receiver’s health and caregiving factors are controlled for.

**Conclusion:**

Older men and women differed in health and care needs. Caregivers, especially those caring for men, were often distressed and remained so through time. These results highlight the need for policies that account for the differential care needs and caregiver profiles of men and women in order to offer targetted and appropriate support.

**Supplementary Information:**

The online version contains supplementary material available at 10.1186/s12877-022-03583-6.

## Background

Family or friend caregivers (henceforth referred to as “caregivers”) are commonly defined as individuals providing unpaid care to family or friends with health-related impairments [1]. With a growing aging population, Canada relies on caregivers to support 70–80% of older persons in the community [2, 3]. Other countries also depend heavily on caregivers, with one in six Americans having cared for someone over 50 in 2020 [4], and up to 80% of long-term care in Europe being performed by family or friend caregivers [5]. Caregivers are often distressed [6], defined broadly as “the overall impact of physical, psychological, social, and financial demands of caregiving” [7]. This jeopardizes the quality of care provided, their own health and capacity to continue caregiving activities, and the health of those under their care (henceforth referred to as “care-receivers”) [8].

Population demographic trends can change the landscape of who will be requiring care, who can provide it, and the distress caregivers experience. One notable trend is the narrowing of the gender gap in life expectancy [9], with the age-adjusted men-to-women mortality ratio in Canada dropping from 1.47 to 1992 to 1.28 in 2012 [10]. Its implication is complex – on the one hand, there may be lower rates of widowhood and greater availability of spousal caregivers for women. On the other, more older men will require support, with the caregiving burden mostly falling onto their spouses who may be frail themselves, limited in capacity, and particularly susceptible to becoming distressed [11–13].

Differences in the health and social challenges older men and women face may also impact their care needs and their caregiver’s experience of distress. Past research has demonstrated important sex and gender differences in mortality and morbidity - in North America, older men have higher rates of mortal conditions (e.g., cardiovascular diseases) and are more likely to be hospitalized or admitted into long-term care facilities [14–16]. In contrast, older women live longer both with and without disability and have more morbid conditions (e.g., arthritis) [15, 16]. Due to widowhood and social expectations, women receive less unpaid care but are often expected to undertake greater caregiving responsibilities, even when they themselves require care [4, 13]. Thus, understanding gender differences in care-receivers’ characteristics, health, and care needs is crucial for identifying caregivers at high risk of distress and for providing targeted interventions to alleviate stress and burden.

Past research on gender differences in caregiver distress have focused almost exclusively on caregiver’s gender [13, 17–20] rather than that of the care-receiver. Among most studies of caregiver distress, care-receiver’s gender is either not reported [21–25], not included in modelling [26–28], or adjusted for without reporting its effect size [29–31]. Only one cross-sectional, population-level study reported descriptively that caregivers of men are more likely distressed than caregivers of women (18.2% versus 9.9%) [14]. Those that included care-receiver’s sex as a covariate in regression modelling also found that caring for men increases the odds of distress, but did not elaborate further [32, 33]. Most existing studies also restrict to patients with specific health profiles (e.g., dementia or cancer), or only examine distress cross-sectionally [26, 33]. To our knowledge, no population-level studies have explored trajectories of caregiver distress longitudinally.

Our study addressed these gaps by using population-level, longitudinal data to examine whether primary caregivers of older men and women differ in their distress trajectories. This study had two main objectives: first, we described and compared the characteristics and health of older men and women receiving care as well as their caregivers’ characteristics and caregiving profile. Second, we described how caregiver distress (at baseline and over time) differed according to the care-receiver’s gender. We hypothesized that caregivers of older men are more likely to be distressed than caregivers of older women. This is at least partly because older men have more health conditions and functional impairments [14], which require greater care. We also expected caregivers of older men to more likely be spouses and co-residing – as women are generally younger and still outlive their husbands – both of which increase the likelihood of distress [26, 32].

## Method

### Data source

This study used population-based data in Ontario, a multicultural and the most populous province in Canada. Since 2002, the Resident Assessment Instrument-Home Care (RAI-HC) has been used by case managers and care coordinators in Ontario to determine the needs of persons expected to be, or currently are, long-term recipients of home care or those applying for admission into a long-term care facility [34]. This standardized, well-validated [35–37], and highly reliable [35–37] assessment is routinely administered to record information on the client’s physical, cognitive, and social functions, service use, and their caregivers’ characteristics and caregiving profile [33]. While all persons assessed by the RAI-HC have additional care needs, not everyone receives home care as some may decline home care or are admitted to other care settings while waiting for home care. Some receive multiple RAI-HC assessments, as existing policies requires a reassessment at least every 6 months or when significant changes in care needs occur [34]. Individual-level RAI-HC data is held and analyzed at ICES, a non-profit research institute funded by the Ontario Ministry of Health and Long-term Care. Its legal status under Ontario’s health information privacy law allows it to collect and analyze personal health data, without consent, for research and health system evaluation.

### Study sample

**Fig. 1:**
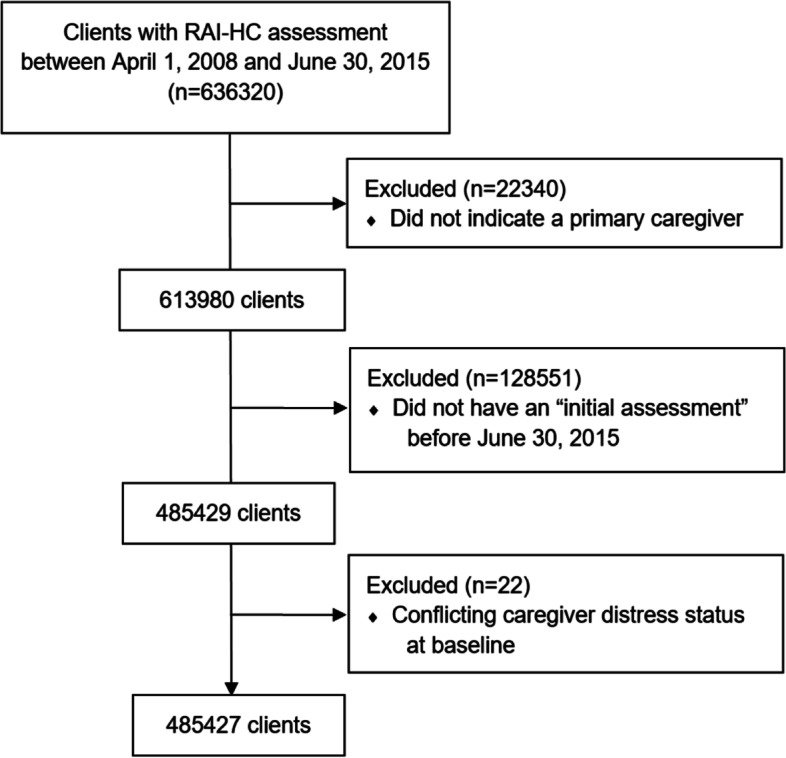
CONSORT flow diagram of the cohort creation

Our study population comprised of adults aged over 50 years and residing in Ontario who have received a RAI-HC “initial assessment” (indicative of a first assessment since opening of a care case) between April 1st, 2008 and June 30th, 2015. Since RAI-HC data was only available up to June 30, 2016 and to ensure that everyone had at least one-year of follow-up, we only included initial assessments that occurred before June 30, 2015. Clients who did not have a primary caregiver were excluded (see Fig. 1 for a CONSORT diagram of study sample selection). Although the caregivers themselves are not followed longitudinally, the clients (i.e., care-receivers) often receive multiple assessments, allowing us to track their caregivers’ distress status over time.

### Defining Caregiver Distress

The primary caregiver is defined as distressed when one or both of the following items on the RAI-HC is checked:

1) A caregiver is unable to continue in caring activities.

2) The primary caregiver expresses feelings of distress, anger or depression.

Both items are coded by assessors based on clinical judgment informed by their observations of the client and their caregivers [38], hence limiting self-report bias. Jointly developed by interRAI and the Canadian Institute for Health Information, this distress indicator measures both feelings of distress and the caregiving situation in distress [39] and has been used by previous studies [26, 33, 38].

Each person’s initial RAI-HC assessment was used to determine their primary caregiver’s baseline distress status. Some did not have another assessment within one year of their initial assessment and were excluded in trajectory analyses. For those with additional assessments, their caregivers followed one of four possible distress trajectories: (1) initially non-distressed caregivers who remained non-distressed in all subsequent assessments; (2) initially non-distressed caregivers who became distressed in a subsequent assessment; (3) initially distressed caregivers who remained distressed in all subsequent assessments; or (4) initially distressed caregivers who became non-distressed in a subsequent assessment.

### The health, care needs, and caregivers of men and women

Most of the differences in men and women’s care needs and their caregivers’ experience of distress likely pertain to differences in socially constructed roles, behaviours, experiences, and identities (i.e., gender-based) rather than to biological, physical, or physiological reasons (i.e., sex-based) [40]. However, as gender is not collected by the RAI-HC assessment, we used care-receiver’s sex (male or female) as a proxy. While all analyses were performed using care-receiver’s sex, terms of gender (e.g., men, women) were used throughout the paper, which is consistent with other studies examining gender using health administrative data [14, 41].

We examined the following factors of older men and women at their initial (i.e., baseline) RAI-HC assessment: demographic characteristics (age, marital status, place of residence, co-residence status); functional status indicated by their self-performance in activities of daily living (ADLs), difficulties in independent activities of daily living (IADL), and continence; cognitive performance and presence of delirium; mood and behavioural issues such as disruptive behaviours and depressive symptoms; and presence of the comorbidities such as dementia and cardiovascular diseases (CVD). We also described their primary caregiver’s relationship to them (“kinship”), whether caregivers co-resided, provided ADL/IADL care and emotional support, and the hours of care provided on an average week.

### Statistical analyses

#### Gender differences in health, care needs, and caregiver profiles

Descriptive analyses (calculations of mean, standard deviations, and proportions) were performed on the socio-demographics, health, care needs, and caregivers of men and women separately, along with tests of statistical significance (t-test, chi-square test) for differences between men and women.

#### Gender differences in caregiver distress

To examine caregivers’ distress trajectories, we calculated the proportions of caregivers who were distressed at baseline, proportions of initially non-distressed caregivers who became distressed, and proportions of initially distressed caregivers who remained distressed. These calculations are performed for men and women separately, and further stratified by type of kinship. We also performed three logistic regressions with baseline caregiver distress as the outcome: Model 1 examined the unadjusted effect of care-receiver’s gender; Model 2 adjusted for well-known predictors of caregiver distress: care-receiver’s age, marital status, place of residence, and caregiver’s kinship and co-residence status [26, 32, 33]; Model 3, in addition to covariates in Model 2, adjusted for the care-receivers’ diagnosis of Alzheimer’s disease or dementia, functional status, behavioural issues, and hours of care provided by their primary caregiver, all of which have also previously been identified as predictors of caregiver distress [19, 26, 31–33, 42]. All covariates included in regressions were pre-specified, and checked for potential collinearity by examining the correlation matrices and cross-tabulation of frequencies as well as the regression parameters for large standard errors. Since no collinearity issues were detected, all were kept in the models.

Since we hypothesized that gender difference in caregiver distress is at least partially due to men having more health and behavioural issues and requiring more care, we expected the effect of sex to decrease after controlling for these factors. Since models 2 and 3 included variables with missing data (with “hours of care” having the highest proportion of missingness at 12.2%), multiple imputation for missing data was performed (number of imputations = 15) using the fully conditional specification method, which uses a separate conditional distribution for each variable and is more appropriate if the imputation involves binary variables [43]. All analyses were performed in SAS Enterprise Guide (V.6.1) [44].

## Results

The RAI-HC assessments of 485,407 older adults, 60.5% of whom women, were included in this study. Table 1 shows the characteristics of these men and women, who their primary caregivers were, and the type and amount of care received as indicated on their baseline assessment. The mean and median age of men were two years less than the age of women. Men were more likely to be married and residing in private homes with their spouse, while women were more often divorced or widowed and living alone or in group setting. Caregivers of men were often their spouses (52.0%), while women were mostly cared by their child/child-in-law (60.1%). Caregivers of men were also more likely to co-reside, provide ADL care, and provide more hours of care.

**Table 1:** Characteristics of older men and women receiving care and their caregivers

**Care-receiver’s demographic and assessment-related characteristics**		**Women Receiving Care **(*n*=293721)	**Men Receiving Care **(*n*=191686)
		**% (n)**^**a**^	**% (n)**
Age	Mean ± SD	79.0 (10.4)	77.1 (10.5)
	Median (IQR^b^)	81.0 (13.0)	79.0 (15.0)
	50 to 64 years	11.7 (34210)	14.8 (28403)
	65 to 79 years	28.5 (83712)	32.6 (62572)
	Over 80 years	59.9 (175799)	52.5 (100711)
Marital status	Married	31.1 (91494)	61.7 (118353)
	Never Married	4.9 (14393)	6.5 (12526)
	Widowed/separated/divorced	62.8 (184325)	30.2 (57903)
	Other	1.2 (3509)	1.5 (2904)
Place of residence at time of referral	Private home with home care	75.3 (221038)	78.0 (149457)
	Private home without home care	13.9 (40916)	14.1 (27027)
	Non-private home	10.8 (31728)	7.9 (15182)
	Missing	<0.1% (39)	<0.1% (20)
Who care-receiver lived with at the time of referral	Lived alone	39.9 (117196)	23.6 (45133)
	Lived with spouse only	24.0 (70456)	48.2 (92358)
	Lived with spouse and others	5.5 (16265)	11.2 (21376)
	Lived with child (not spouse)	17.7 (51896)	6.9 (13240)
	Lived with others/in group setting	12.9 (37869)	10.2 (19559)
	Missing	<0.1 (39)	<0.1 (20)
**Characteristics of the primary caregiver and care provided**			
Caregiver lived with care-receiver	Yes	47.4 (139338)	65.6 (125735)
Caregiver's relationship to care-receiver	Child/child-in-law	60.1 (176380)	32.3 (62000)
	Spouse	23.5 (69080)	52.0 (99654)
	Other relatives	10.2 (29812)	9.1 (17514)
	Friend/neighbor	6.3 (18449)	6.5 (12518)
Caregiver provides emotional support^c^	Yes	97.4 (286209)	97.3 (186545)
Caregiver provides care for instrumental activities of daily living (IADL)^d^	Yes	89.6 (263090)	89.3 (171202)
Caregiver provides care for activities of daily living (ADL)	Yes	38.0 (111568)	47.5 (91125)
Hours of care provided for ADL and IADL activities in the last 7 days	10 or fewer hours	42.7 (125475)	33.0 (63243)
	11-20 hours	19.4 (56955)	19.5 (37440)
	21+ hours	26.2 (76848)	34.6 (66297)
	missing	11.7 (34443)	12.9 (24706)

^a^All *p*-values for differences between women and men care-receivers are <0.0001 unlessunless otherwise specified

^b^IQR: inter-quartile range

^c^Difference between women and men care-receivers are significant at *p* = 0.0078

^d^Difference between women and men care-receivers are significant at *p* = 0.0043

Table 2 shows the health and functioning of older men and women. In general, men receiving care had poorer health and greater care needs. Though the performance of men and women in ADL tasks were similar, more men had great difficulty in two or all three IADLs. While more women had possible depression (18.3% vs. 15.0% of men), incontinence (43.8% vs. 34.1% of men), and fractures (15.3% vs. 8.4% of men), men had slightly greater cognitive impairment (indicated by higher Cognitive Performance Scale (CPS) scores), were more likely to exhibit behavioural issues and delirium, and were more likely to have cardiovascular diseases, Parkinson’s disease, and cancer. The prevalence of Alzheimer’s/dementia were similar between men and women.

**Table 2:** Health, functional status, and behavioural symptoms of men and women

		**Women Receiving Care**	**Men Receiving Care**
**Functional status**		**% (n)**^**a**^	**% (n)**
ADL self-performance hierarchy	0: Independent	61.7 (181175)	57.1 (109518)
	1: Supervision required	10.6 (31051)	11.9 (22843)
	2: Limited impairment	13.2 (38809)	14.4 (27628)
	3: Extensive assistance required (I)	5.2 (15153)	6.9 (13256)
	4: Extensive assistance required (II)	5.0 (14594)	5.1 (9805)
	5: Dependent	3.6 (10441)	3.6 (6829)
	6: Total Dependence	0.8 (2498)	0.9 (1807)
IADL difficulty scale	0: No difficulty in any IADLs	5.1 (14908)	5.8 (11207)
	1: Some difficulty in one IADL	7.7 (22591)	5.4 (10328)
	2: Some difficulty in two IADLs	14.3 (41888)	11.7 (22484)
	3: Some difficulty in all three IADLs	2.1 (6102)	2.0 (3803)
	4: Great difficulty in one IADL	20.7 (60673)	17.2 (33024)
	5: Great difficulty in two IADLs	37.0 (108645)	40.4 (77526)
	6: Great difficulty in all three IADLs	13.2 (38914)	17.4 (33314)
Bladder/bowel incontinence	Incontinence present in last 7 days	43.8 (128695)	34.1 (65409)
Pain intensity	Mild or no pain	49.3 (144917)	59.1 (113302)
	Moderate	36.2 (106253)	30.1 (57703)
	Severe	11.3 (33100)	8.4 (16151)
	Pain is horrible	3.2 (9451)	2.4 (4530)
**Cognitive abilities**			
Cognitive Performance Scale	0: Intact	44.4 (130370)	41.0 (78515)
	1: Borderline intact	17.0 (48145)	16.4 (31435)
	2: Mild impairment	16.2 (47686)	16.9 (32374)
	3: Moderate impairment	18.5 (54266)	20.2 (38756)
	4: Moderately severe impairment	1.4 (4107)	2.0 (3914)
	5: Severe impairment	2.5 (7300)	2.8 (5447)
	6: Very severe impairment	0.6 (1847)	0.7 (1245)
Had delirium in the last 90 days	Yes	5.8 (17005)	7.3 (13919)
**Mood and behavior**			
Wandering behavior	Yes	2.4 (7143)	3.2 (6101)
Verbally and/or physically abusive	Yes	2.8 (8180)	4.6 (8738)
Socially inappropriate/disruptive	Yes	1.5 (4400)	2.1 (3992)
Depression rating scale (categorized)	Possible depression	18.3 (53651)	15.0 (28786)
**Comorbidities**			
Has Alzheimer's or dementia	Yes	21.0 (61716)	21.7 (41534)
Has any cardiovascular diseases^b^	Yes	36.6 (107448)	47.0 (90140)
Has Parkinson’s disease	Yes	2.4 (6978)	5.1 (9726)
Has any fractures	Yes	15.3 (45009)	8.4 (16059)
Has cancer (excluding skin cancer)	Yes	14.5 (42625)	21.9 (41900)

^a^All *p*-values for differences between women and men care-receivers are <0.0001

^b^Includes stroke, coronary heart diseases, congestive heart failure, or peripheral vascular disease

**Fig. 2:**
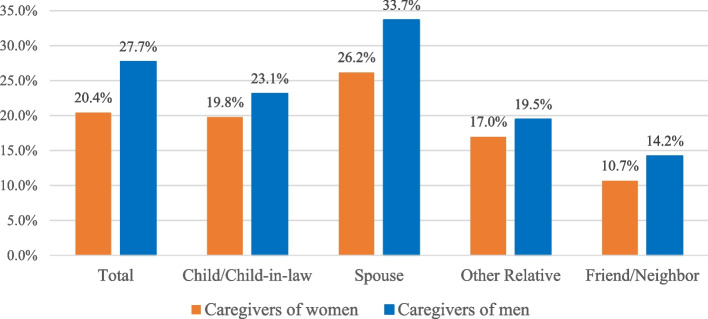
Proportions of caregivers of men and women who were distressed at baseline, by kinship to care-receivers. Note: all differences between caregivers of men and women were statistically significant at p<0.0001

Figure 2 shows the proportions of caregivers of men and women, stratified by kinships, who were distressed at baseline. Spousal caregivers were most likely to be distressed, followed by child/child-in-law, while friends or neighbors providing care were least likely to be distressed. More caregivers of men were distressed at baseline (27.7% of all versus 20.4% caregivers of women). This gender difference is present across all types of kinships, but is especially prominent for spouses (33.7% of spousal caregivers of men vs. 26.2% of spousal caregivers of women).

**Fig. 3:**
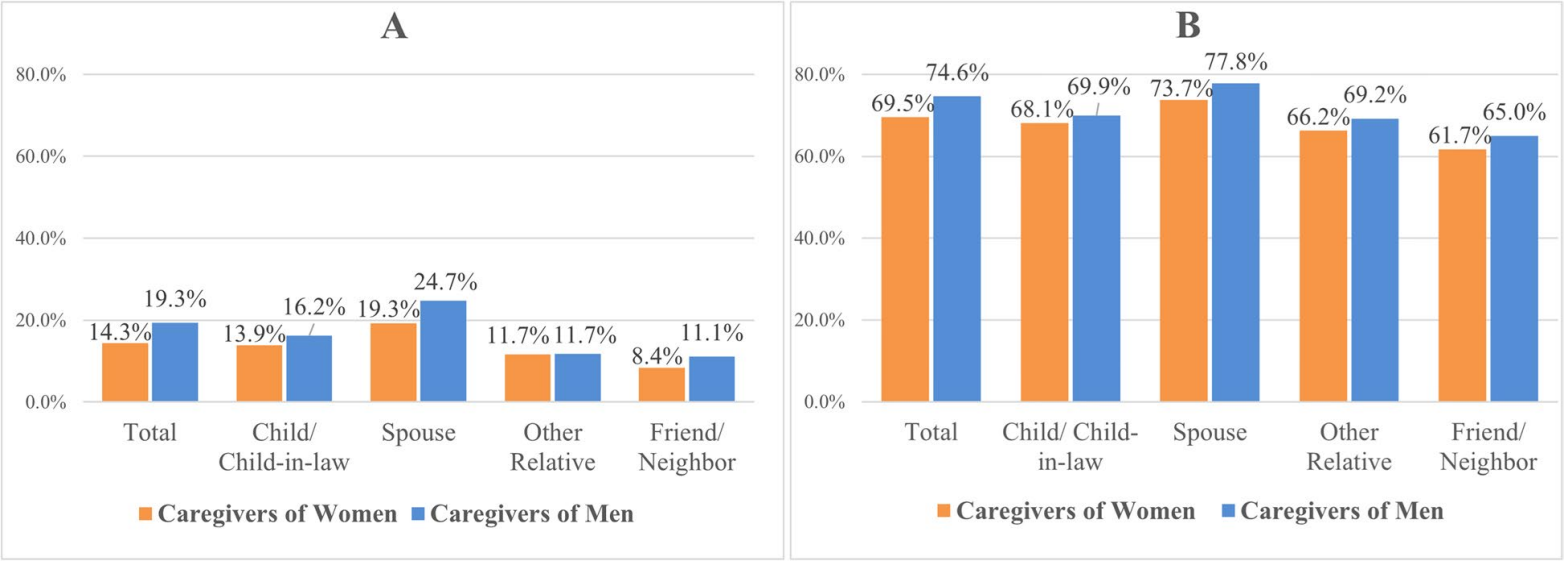
**A** Proportions of initially non-distressed caregivers who became distressed, and (**B**) initially distressed caregivers who remained distressed, by care-receiver’s gender and caregiver’s kinship. Note: all differences between caregivers of men and women were statistically significant at *p* < 0.0001, except for caregivers who were other relatives in Fig. 3A

Most older women (53.7%) and men (56.0%) did not have another RAI-HC assessment within one-year of their initial assessment, hence excluding them from distress trajectory analyses. Among those with multiple assessments, 22.6% and 31.7% of caregivers of women and men, respectively, were distressed at baseline. In addition, Fig. 3 shows that initially non-distressed caregivers of men were more likely to become distressed (19.3% versus 14.3% of initially non-distressed caregivers of women), and initially distressed caregivers of men were also more likely to remain distressed (74.6% versus 69.5% of initially distressed caregivers of women). These gender differences were present across almost all types of kinship, except for distant relatives where there were no sex differences in the one-year incidence of becoming distressed. The gender difference is more prominent among spousal caregivers, who also had the highest proportions of becoming or remaining distressed.

Table 3 shows the odds ratios (ORs) and 95% confidence intervals (CIs) of the three logistic regression models. Looking at the unadjusted effect of care-receiver’s gender in Model 1, caregivers of men had 1.49 (CI: 1.47–1.51) times the odds of being distressed at baseline than caregivers of women. The OR of gender decreased to 1.26 (CI: 1.24–1.28) after controlling for care-receiver’s characteristics and caregiver’s kinship and co-residence status in Model 2, and further decreased to 1.17 (CI: 1.15–1.19) in Model 3 after also controlling for care-receiver’s health, functional status, behavioural issues, and the hours of care their caregivers provided. Though the effect of gender remains significant in Model 3, other factors have much larger independent effects on caregiver distress. Care-receiver’s IADL and CPS scores had the largest impacts, more than doubling the odds of caregiver distress if the care-receiver had some IADL difficulty or moderate to very severe cognitive impairment (indicated by scores above three). Caregivers who provided more than 20 h of care per week also doubled their odds of being distressed (OR = 2.23, CI: 2.18–2.28). In contrast, they were half as likely to be distressed if their care-receivers resided in a congregate setting instead of a private home. In addition, caregivers who were spouses, children, or relatives of their care-receivers were slightly more distressed than if they were a friend or neighbor. Co-residence also increased the odds of distress, but its OR decreased from 1.75 in Model 2 to a range of 1.17 in Model 3 after controlling for hours of care provided and care-receiver’s health and behavioural issues.

**Table 3:** Logistic regression models with baseline caregiver distress as outcome

		Model 1	Model 2	Model 3
		**OR (95% CI)**		
**Care-receiver’s gender**	Men vs. Women	1.49 (1.47–1.51)	1.26 (1.24 1.28)	1.17 (1.15–1.19)
**Care-receiver’s age** (reference: 50 to 64)	65 to 79		1.52 (1.48–1.56)	1.15 (1.12–1.19)
	Over 80		1.95 (1.91-2.00)	1.20 (1.17–1.23)
**Marital status** (reference: never married)	Married		1.14 (1.10–1.19)	1.36 (1.30–1.42)
	Widowed/separated/divorced		0.79 (0.76–0.83)	0.89 (0.86–0.93)
**Place of residence** (reference: private home)	Board care, assisted living, or group home		0.80 (0.78–0.83)	0.54 (0.58 − 0.56)
	Residential care facilities		0.74 (0.70–0.78)	0.46 (0.44–0.49)
**Caregiver co-resides**	Yes vs. No		1.75 (1.72–1.78)	1.17 (1.15–1.20)
**Caregiver’s relationship** **to care-receiver** (reference: friend/neighbor)	Spouse		1.53 (1.46–1.60)	1.30 (1.24–1.36)
	Child/child-in-law		1.61 (1.55–1.67)	1.33 (1.27–1.38)
	Other relative		1.55 (1.48–1.61)	1.36 (1.30–1.43)
**ADL self-performance hierarchy scale** (reference: 0)	1			1.30 (1.28–1.33)
	2			1.28 (1.26–1.30)
	3			1.38 (1.34–1.41)
	4			1.33 (1.29–1.38)
	5			1.26 (1.21–1.32)
	6			1.14 (1.11–1.17)
**IADL difficulty scale** (reference: 0)	1			1.25 (1.17–1.34)
	2			1.70 (1.61–1.79)
	3			2.23 (2.20–2.23)
	4			2.20 (1.90–2.21)
	5			2.45 (2.20–2.50)
	6			2.56 (2.33–2.79)
**Cognitive performance scale** (reference: 0)	1			1.68 (1.66–1.71)
	2			1.94 (1.90–1.98)
	3			2.43 (2.37–2.48)
	4			2.40 (2.29–2.51)
	5			2.38 (2.29–2.47)
	6			2.53 (2.28–2.82)
**Wandering**	Yes vs. No			1.42 (1.37–1.48)
**Verbally/physically abusive**	Yes vs. No			1.42 (1.28–1.47)
**Socially inappropriate or disruptive behaviour**	Yes vs. No			1.27 (1.21–1.33)
**Resists care**	Yes vs. No			1.37 (1.33–1.41)
**Bladder/bowel incontinence**	Incontinent vs. Continent			1.25 (1.23–1.27)
**Has Alzheimer’s/dementia**	Yes vs. No			1.35 (1.30–1.40)
**Pain intensity** (reference: mild or no pain)	Moderate			1.02 (0.99–1.04)
	Severe			1.20 (1.18–1.22)
	Pain is horrible			1.48 (1.43–1.54)
**Hours of care per week** (reference: fewer than 10)	10 to 20 h			1.66 (1.63–1.69)
	21 h or more			2.23 (2.18–2.28)

## Discussion

Our study showed that older men and women receiving care in Ontario differed in demographic characteristics, health, care needs, and caregiver profiles. We also showed that caregiver distress is substantial, regardless of who the caregiver or care-receiver is. Not only were approximately one quarter of caregivers distressed at baseline, more than 70% of these initially distressed caregivers also remained distressed throughout the year. Among initially non-distressed caregivers, about 16% subsequently became distressed within one year.

As hypothesized, caregivers of men and women had different levels of distress: across all types of kinship, caregivers of men were more likely to be distressed, remain distressed, or become distressed. This is partly because of differences in their demographic profiles and levels of disability and need. Similar to findings from other studies in Canada [10, 14, 45], men had more functional and cognitive impairments, behavioural issues and required more care, all of which significantly increased the odds of caregiver distressed as shown in our regression analyses and in past findings [26, 29, 32, 33]. The effect of care-receiver’s gender on caregiver distress also decreased after these factors were controlled for, further confirming that the effect of gender is largely attributed to differences in health and care profiles. This explanation also aligns with past studies which have consistently shown that having complex health conditions increases caregiving burden and financial strain, leading to greater caregiver stress [4, 46, 47]. Additionally, more women than men lived in congregate settings which greatly reduced the odds of caregiver distress. This factor is often neglected in past studies, but there is evidence that the social and professional supports provided through congregate settings could reduce caregivers’ stress and burnout [48–50].

Caregivers of older men and women also differed in kinship and co-residence status. Women were mostly (60%) cared for by their children or children-in-laws while men were mostly (52%) cared by their spouses. This is likely because, as shown in Table 1, women were more likely widowed and older, and therefore had no spouses or frail or institutionalized spouses. Spousal caregivers, being older themselves and often feeling obligated to undertake caregiving responsibilities [18], have been shown to experience more depression symptoms and greater financial and physical strain [4, 18, 51]. Spouses of men are also mostly women since most men are in heterosexual relationships, and women caregivers often experience more negative consequences of caregiving such as employment/financial repercussions [52–54], difficulty juggling child-rearing and caregiving responsibilities [55], and social isolation [56]. In addition, men were more likely to live with their caregivers, and co-residence has been associated with higher distress, strain, and negative health consequences [18, 29, 32] as the constant caregiving burden can be emotionally and physically draining. In our regression analyses, co-residence indeed increased the odds of caregiver distress, but the magnitude of its effect decreased substantially after adjusting for hours of care provided and care-receivers’ health and behavioural issues. This is unsurprising as the decision to co-reside is often driven by care-receivers having complex health issues and greater care needs [51].

It is interesting to note that the OR of gender was substantially, but only partially, reduced after controlling for other factors. The remainder of its effect on caregiver distress may be due to residual confounding, the principal ones being caregiver’s age and gender, which we do not have data on. There may also be multi-way interactions between types of kinship, caregiver’s gender, and care-receiver’s gender, which we also could not test due to the lack of data. For example, Lott (1991) found that women caregivers were more burdened if they cared for a mother or mother-in-law than a father or father-in-law, while men caregivers were more burdened caring for a parent than for a parent-in-law [57]. Obtaining data on all the multitude of contributors of caregiver distress for modelling has always been challenging for researchers. Care-receiver’s gender, however, can be easily measured or estimated from population distribution. By highlighting how men and women differ in their health, behaviour, and caregiving/caregiver profiles and providing the unadjusted and partially-adjusted effects of care-receiver’s gender on caregiver distress, we demonstrate the importance of including care-receiver’s gender when modelling caregiver distress, especially when information on factors such as functioning and behaviours are not available.

This study has several strengths. First, using routinely collected population-level data results in better generalizability and less selection bias. Unlike other studies that only examined caregiver distress cross-sectionally, we followed care-receivers longitudinally for their caregivers’ distress trajectories. Finally, we not only described but also investigated reasons for gender differences caregiver distress by looking at changes in the OR of care-receiver’s gender when health, care needs, and caregiving factors are added to the regression model.

### Limitations

Our research is constrained by several limitations commonly associated with using health administrative data. First, the RAI-HC is limited in variable availability. Due to the lack of gender variables, we resorted to using care-receiver’s sex as a proxy for gender. This ignores other gender identities such as being transgendered or gender-fluid, and prevents us from identifying or separating gender-based versus sex-based differences. Other confounders or determinants of caregiver distress, such as caregiver’s gender and age, were also unavailable. Furthermore, caregiver distress was defined as a binary variable since information on the degree or nuanced aspects of distress were unavailable.

Another limitation is that we could only follow the trajectories of caregivers through their care-receivers’ assessments, as the RAI-HC does not collect identification information of the caregivers. Thus, we could not ensure that each care-receiver had the same caregiver throughout their multiple assessments. This is partially mitigated by limiting the follow-up period to one year, during which a change of the primary caregiver is expected to be infrequent. We verified this assumption by showing that the caregiver’s kinship to the care-receiver changed for only 2.8% of our cohort. This assumption is also supported by a national survey in the U.S. which found that 85% of surveyed caregivers had provided care for more than one year [58], as well as an AARP report stating that caregivers of adults provided on average 4.5 years of care [4].

Finally, more than half of our cohort did not have the requisite multiple assessments within one year to measure distress trajectory. This might result in selection bias, as those with multiple assessments are more likely to have change in their health status, and thus our trajectory findings may describe more unstable care-receivers. Selection bias may also have occurred as certain disadvantaged groups (e.g., immigrants with language barriers) have less access to or awareness of health care resources, and thus are both less likely to receive RAI-HC assessments (i.e., less represented in our cohort) and more likely to have distressed caregivers.

Past studies on sex differences in distress have often been challenged on the possibility that women caregivers simply report more distress while men are reluctant to do so despite being similarly distressed. This is unlikely in our study since the two items used to define caregiver distress were evaluated by assessors based on reports from the caregivers and clients as well as the assessors’ observations and clinical judgement of the client’s situation, including projection of future needs [59].

## Conclusion

Our study showed that, across all types of kinship, caregivers of men were more likely to be distressed, remain distressed or become distressed within one year. This is partly because older men had more health, functional, and behaviour issues which required more care, and partly because their caregivers are more likely to be spouses and co-residing. Policy makers and health systems planners should account for the different care needs of men and women and the different profiles of their caregivers in order to provide adequate and appropriate resources and support. Our study also highlights the need to examine trajectories of caregiver distress longitudinally and on a population level, as consequences of prolonged or changes in distress on caregivers, care-receivers, and the healthcare system remains unclear.

## Supplementary Information

**Additional file 1:** **Supplementary Table 1.** Characteristics of older men and women receivingcare and their caregivers. **Supplementary Table 2.** Health, functionalstatus, and behavioural symptoms of men and women. 

## Data Availability

The data that support the findings of this study are available from ICES but restrictions apply to the availability of these data, which were used under license for the current study, and so are not publicly available. Data are available only by request through ICES.

## Abbreviations

ADL: Activities of daily living
CI: Confidence interval
CPS: Cognitive performance scale
CVD: Cardiovascular diseases (including stroke, coronary heart diseases, and congestive heart failure)
DRS: Depression rating scale
IADL: Instrumental activities of daily living
IQR: inter-quartile range
OR: Odds ratio
RAI-HC: Resident assessment instrument for home care
SD: Standard deviation

## References

[1] Stall NM, Campbell A, Reddy M, Rochon PA (2019). Words Matter: The Language of Family Caregiving. J Am Geriatr Soc.

[2] Hébert R, Dubois MF, Wolfson C, Chambers L, Cohen C. Journals Gerontol - Ser A Biol Sci Med Sci. 2001;56(11):693–9. 10.1093/gerona/56.11.M693. 10.1093/gerona/56.11.m693 11682577

[3] Carriere Y, Belanger A, Lafreniere S (2003). Dependent seniors at home-formal and informal help. Heal Rep.

[4] AARP. Caregiving in the U.S. 2020: A Focused Look at Family Caregivers of Adults Age 50+.; 2020. https://www.caregiving.org/wp-content/uploads/2021/05/AARP1340_RR_Caregiving50Plus_508.pdf.

[5] Rodrigues R, Hoffman F. Informal Carers: Who Takes Care of Them? Policy Brief, April 2010.; 2010. file:///C:/Users/LWS/Downloads/Informal_Carers_Who_Takes_Care_of_Them.pdf.

[6] Taylor MG, Quesnel-Vallée A (2017). The structural burden of caregiving: Shared challenges in the United States and Canada. Gerontologist.

[7] George LK, Gwyther LP (1986). Caregiver well-being: a multidimensional examination of family caregivers of demented adults. George LK ed Gerontologist..

[8] Cooney C, Howard R, Lawlor B (2006). Abuse of vulnerable people with dementia by their carers: can we identify those most at risk?. Int J Geriatr Psychiatry.

[9] OECD. Health at a Glance 2017: OECD Indicators - Life Expectancy by Sex and Education Level. OECD Publishing; 2017. 10.1787/health_glance-2017-en.

[10] Rosella LC, Calzavara A, Frank JW, Fitzpatrick T, Donnelly PD, Henry D (2016). Narrowing mortality gap between men and women over two decades: A registry-based study in Ontario, Canada. BMJ Open.

[11] Ornstein KA, Wolff JL, Bollens-Lund E, Rahman O-K, Kelley AS (2019). Spousal Caregivers Are Caregiving Alone In The Last Years Of Life. Health Aff.

[12] Potier F, Degryse J-M, Aubouy G (2018). Spousal Caregiving is Associated With an Increased Risk of Frailty: A Case-Control Study. J Frailty Aging.

[13] Sharma N, Chakrabarti S, Grover S (2016). Gender differences in caregiving among family - caregivers of people with mental illnesses. World J psychiatry.

[14] Gruneir A, Forrester J, Camacho X, Gill SS, Bronskill SE (2013). Gender differences in home care clients and admission to long-term care in Ontario, Canada: A population-based retrospective cohort study. BMC Geriatr.

[15] Crimmins EM, Kim JK, Hagedorn A (2002). Life with and without disease: women experience more of both. J Women Aging.

[16] Bélanger A, Martel L, Berthelot J-M, Wilkins R (2002). Gender differences in disability-free life expectancy for selected risk factors and chronic conditions in Canada. J Women Aging.

[17] Pickard L, Wittenberg R, Comas-Herrera A, King D, Malley J (2007). Care by Spouses, Care by Children: Projections of Informal Care for Older People in England to 2031. Soc Policy Soc.

[18] Pinquart M, Sörensen S, Spouses (2011). Adult Children, and Children-in-Law as Caregivers of Older Adults: A Meta-Analytic Comparison. Psychol Aging.

[19] Stall NM, Kim SJ, Hardacre KA (2019). Association of Informal Caregiver Distress with Health Outcomes of Community-Dwelling Dementia Care Recipients: A Systematic Review. J Am Geriatr Soc.

[20] Xiong C, Biscardi M, Astell A (2020). Sex and gender differences in caregiving burden experienced by family caregivers of persons with dementia: A systematic review. McMunn A. ed PLoS One.

[21] Sherwood PR, Given CW, Given BA, Von Eye A (2005). Caregiver burden and depressive symptoms: Analysis of common outcomes in caregivers of elderly patients. J Aging Health.

[22] Ducharme F, Lévesque L, Lachance L, Kergoat M-J, Coulombe R (2011). Challenges associated with transition to caregiver role following diagnostic disclosure of Alzheimer disease: a descriptive study. Int J Nurs Stud.

[23] Lee Y, Xu L, Kim BJ, Chen L (2020). Leisure activity, gender and depressive symptoms among dementia caregivers: findings from the REACH II. Aging Ment Health.

[24] Papastavrou E, Kalokerinou A, Papacostas SS, Tsangari H, Sourtzi P (2007). Caring for a relative with dementia: family caregiver burden. J Adv Nurs.

[25] Takai M, Takahashi M, Iwamitsu Y, Oishi S, Miyaoka H (2011). Subjective experiences of family caregivers of patients with dementia as predictive factors of quality of life. Psychogeriatrics.

[26] Hirdes JP, Freeman S, Smith TF, Stolee P (2012). Predictors of caregiver distress among palliative home care clients in Ontario: Evidence based on the interRAI Palliative Care. Palliat Support Care.

[27] Chappell NL, Dujela C, Smith A (2014). Caregiver Well-Being: Intersections of Relationship and Gender. Res Aging.

[28] Pöysti MM, Laakkonen ML, Strandberg T, et al. Gender Differences in Dementia Spousal Caregiving. Int J Alzheimers Dis. 2012;2012. 10.1155/2012/162960. 10.1155/2012/162960 PMC3465980 23056990

[29] Chong AML, Kwan CW, Lou VWQ, Chi I (2017). Can domestic helpers moderate distress of offspring caregivers of cognitively impaired older adults?. Aging Ment Heal.

[30] Chisholm A. Understanding Caregiver Burden and Hospital Use Among Older Home Care Recipients in Nova Scotia. Published online 2017:1–93. https://dalspace.library.dal.ca/bitstream/handle/10222/73534/Chisholm_Ashley_MSc_CHE_Dec_2017.pdf?sequence=3&isAllowed=y.

[31] Chang BW, Hirdes JP. A Cross-Sectional Study to Compare Caregiver Distress Among Korean Canadian, Chinese Canadian, and Other Canadian Home Care Clients. SAGE Open. 2015;5(2). 10.1177/2158244015591824.

[32] Mitchell LA, Hirdes J, Poss JW, Slegers-Boyd C, Caldarelli H, Martin L (2015). Informal caregivers of clients with neurological conditions: profiles, patterns and risk factors for distress from a home care prevalence study. BMC Health Serv Res.

[33] Canadian Institute for Health Information. Supporting Informal Caregivers — The Heart of Home Care Executive Summary.; 2010. https://secure.cihi.ca/free_products/Caregiver_Distress_AIB_2010_EN.pdf.

[34] Ministry of Health and Long-Term Care (2006). Community Care Access Centres: Client Services Policy Manual. Ministry of Health and Long-Term Care.

[35] Kwan C-W, Chi I, Lam T-P, Lam K-F, Chou K-L (2000). Validation of Minimum Data Set for Home Care Assessment Instrument (MDS-HC) for Hong Kong Chinese Elders. Clin Gerontol.

[36] Morris JN, Fries BE, Steel K (1997). Comprehensive clinical assessment in community setting: applicability of the MDS-HC. J Am Geriatr Soc.

[37] Landi F, Tua E, Onder G (2000). Minimum data set for home care: a valid instrument to assess frail older people living in the community. Med Care.

[38] Betini RSD, Hirdes JP, Lero DS, Cadell S, Poss J, Heckman G (2017). A longitudinal study looking at and beyond care recipient health as a predictor of long term care home admission. BMC Health Serv Res.

[39] Health Quality Ontario. The Reality of Caring: Distress among the Caregivers of Home Care Patients. 111; 2016. http://www.hqontario.ca/Portals/0/documents/system-performance/reality-caring-report-en.pdf.

[40] Canadian Institute of Health Research. How to integrate sex and gender into research. Published 2019. https://cihr-irsc.gc.ca/e/50836.html Accessed 20 Apr 2021.

[41] Gruneir A, Bronskill SE, Maxwell CJ (2016). The association between multimorbidity and hospitalization is modified by individual demographics and physician continuity of care: a retrospective cohort study. BMC Health Serv Res.

[42] Covinsky KE, Newcomer R, Fox P (2003). Patient and caregiver characteristics associated with depression in caregivers of patients with dementia. J Gen Intern Med.

[43] Liu Y, De A (2015). Multiple Imputation by Fully Conditional Specification for Dealing with Missing Data in a Large Epidemiologic Study. Int J Stat Med Res.

[44] SAS®. Enterprise Guide 8.1 [program]. Published online 2019.

[45] Canadian Institute for Health Information. Health Care in Canada, 2011 A Focus on Seniors and Aging. 2011. https://www.homecareontario.ca/docs/default-source/publications-mo/hcic_2011_seniors_report_en.pdf.

[46] Bevans M, Sternberg EM (2012). Caregiving burden, stress, and health effects among family caregivers of adult cancer patients. JAMA.

[47] Reinhard SC, Young HM, Levine C, Kelly K, Choula RB, Accius J. Home Alone Revisited: Family Caregivers Providing Complex Care. 2019. https://www.aarp.org/content/dam/aarp/ppi/2019/04/home-alone-revisited-family-caregivers-providing-complex-care.pdf.

[48] Anderson S, Parmar J, Dobbs B, Tian PGJ (2021). A Tale of Two Solitudes: Loneliness and Anxiety of Family Caregivers Caring in Community Homes and Congregate Care. Int J Environ Res Public Health.

[49] Smit D, te Boekhorst S, de Lange J, Depla MFIA, Eefsting JA, Pot AM (2011). The long-term effect of group living homes versus regular nursing homes for people with dementia on psychological distress of informal caregivers. Aging Ment Health.

[50] Brodaty H, Hadzi-Pavlovic D (1990). Psychosocial effects on carers of living with persons with dementia. Aust N Z J Psychiatry.

[51] Hounsell C, Jed Johnson W, Seals Carol Levine E, et al. Caregiving in the U.S. – AARP 2015 Report. 2015;81.

[52] Lero D, Joseph G. A Systematic Review of The Literature on Combining Work and Eldercare in Canada. 2007. https://www.familycaregiversbc.ca/wp-content/uploads/2015/04/systemic-review-of-combining-work-and-elder-care-final-report.pdf.

[53] Brink S (2004). Elder Care the Nexus for Family.

[54] Metlife Mature Market Institute (2011). The MetLife Study of Caregiving Costs to Caregivers The MetLife Study of Caregiving Costs to Working Caregivers Double Jeopardy for Baby Boomers Caring for Their Parents.

[55] Duxbury LE. Balancing Paid Work and Caregiving Responsibilities a Closer Look at Family Caregivers in Canada. (Higgins CA, Schroeder B, editors). Canadian Policy Research Networks]; 2009.

[56] Healthy Aging and Wellness Working Group. Healthy Aging in Canada: A New Vision., Vital Investment A. Published online 2006:21. http://www.phac-aspc.gc.ca/seniors-aines/alt-formats/pdf/publications/public/healthy-sante/vision/vision-eng.pdf.

[57] Lott WC. Adult children’s attitudes toward eldercare: Does consanguinity make a difference? Published online 1991.

[58] of Sciences Engineering, Medicine. Families Caring for an Aging America. (Schulz R, Eden J, editors). The National Academies Press. 2016. doi:10.17226/23606. 27905704

[59] Morris JN, Fries BE, Bernabei R (2010). RAI-Home Care (RAI-HC) User’s Manual.

