# Supplementary material for: Caring for older men and women: whose caregivers are more distressed? A population-based retrospective cohort study
Source: BMC Geriatr. 2022 Nov 22;22:890. doi: 10.1186/s12877-022-03583-6 (PMC9682689; doi:10.1186/s12877-022-03583-6)
Supplement: Supplementary file 1 — Additional file 1: Supplementary Table 1. Characteristics of older men and women receivingcare and their caregivers. Supplementary Table 2. Health, functionalstatus, and behavioural symptoms of men and women. [file 12877_2022_3583_MOESM1_ESM.docx]

**Supplementary Tables**

**Supplementary Table 1** - Characteristics of older men and women receiving care and their caregivers

| **Care-receiver’s demographic and assessment-related characteristics** | | **Women Receiving Care** (n=293721) | **Men Receiving Care** (n=191686) | **Total Cohort**  (n=485407) |  |
| --- | --- | --- | --- | --- | --- |
|  |  | **% (n)** ^[[1]](#footnote-1)^ | **% (n)** | **% (n)** |  |
| Age | Mean ± SD | 79.0 (10.4) | 77.1 (10.5) | 81.6 (8.8) |  |
|  | Median (IQR^[[2]](#footnote-2)^) | 81.0 (13.0) | 79.0 (15.0) | 83.0 (11.0) |  |
|  | 50 to 64 years | 11.7 (34210) | 14.8 (28403) | 12.9 (62613) |  |
|  | 65 to 79 years | 28.5 (83712) | 32.6 (62572) | 30.1 (146284) |  |
|  | Over 80 years | 59.9 (175799) | 52.5 (100711) | 57.0 (276510) |  |
| Marital status | Married | 31.1 (91494) | 61.7 (118353) | 43.2 (209847) |  |
|  | Never Married | 4.9 (14393) | 6.5 (12526) | 5.5 (26919) |  |
|  | Widowed/separated/divorced | 62.8 (184325) | 30.2 (57903) | 49.9 (242228) |  |
|  | Other | 1.2 (3509) | 1.5 (2904) | 1.3 (6413) |  |
| Place of residence at time of referral | Private home with home care | 75.3 (221038) | 78.0 (149457) | 76.3 (370495) |  |
|  | Private home without home care | 13.9 (40916) | 14.1 (27027) | 14.0 (67943) |  |
|  | Non-private home | 10.8 (31728) | 7.9 (15182) | 9.7 (46910) |  |
|  | Missing | <0.1% (39) | <0.1% (20) | <0.1% (59) |  |
| Who care-receiver lived with at the time of referral | Lived alone | 39.9 (117196) | 23.6 (45133) | 33.4 (162329) |  |
|  | Lived with spouse only | 24.0 (70456) | 48.2 (92358) | 33.5 (162814) |  |
|  | Lived with spouse and others | 5.5 (16265) | 11.2 (21376) | 7.8 (37641) |  |
|  | Lived with child (not spouse) | 17.7 (51896) | 6.9 (13240) | 13.4 (65136) |  |
|  | Lived with others/in group setting | 12.9 (37869) | 10.2 (19559) | 11.8 (57428) |  |
|  | Missing | <0.1 (39) | <0.1 (20) | <0.1 (59) |  |
| **Characteristics of the primary caregiver and care provided** | |  |  |  |  |
| Caregiver lived with care-receiver | Yes | 47.4 (139338) | 65.6 (125735) | 54.6 (265073) |  |
| Caregiver's relationship to care-receiver | Child/child-in-law | 60.1 (176380) | 32.3 (62000) | 49.1 (238380) |  |
|  | Spouse | 23.5 (69080) | 52.0 (99654) | 34.8 (168734) |  |
|  | Other relatives | 10.2 (29812) | 9.1 (17514) | 9.7 (47326) |  |
|  | Friend/neighbor | 6.3 (18449) | 6.5 (12518) | 6.4 (30967) |  |
| Caregiver provides emotional support^[[3]](#footnote-3)^ | Yes | 97.4 (286209) | 97.3 (186545) | 97.4 (472754) |  |
| Caregiver provides care for instrumental activities of daily living (IADL)^[[4]](#footnote-4)^ | Yes | 89.6 (263090) | 89.3 (171202) | 89.5 (434292) |  |
| Caregiver provides care for activities of daily living (ADL) | Yes | 38.0 (111568) | 47.5 (91125) | 41.8 (202693) |  |
| Hours of care provided for ADL and IADL activities in the last 7 days | 10 or fewer hours | 42.7 (125475) | 33.0 (63243) | 38.9 (188718) |  |
|  | 11-20 hours | 19.4 (56955) | 19.5 (37440) | 19.4 (94395) |  |
|  | 21+ hours | 26.2 (76848) | 34.6 (66297) | 29.5 (143145) |  |
|  | missing | 11.7 (34443) | 12.9 (24706) | 12.2 (59149) |  |

**Supplementary Table 2.** Health, functional status, and behavioural symptoms of men and women

|  | | **Women Receiving Care**  (n=293721) | **Men Receiving Care**  (n=191686) | Total Cohort  (n= 485407) |
| --- | --- | --- | --- | --- |
| **Functional status** | | **% (n)**^[[5]](#footnote-5)^ | **% (n)** | **% (n)** |
| ADL self-performance hierarchy | 0: Independent | 61.7 (181175) | 57.1 (109518) | 59.9 (290693) |
|  | 1: Supervision required | 10.6 (31051) | 11.9 (22843) | 11.1 (53894) |
|  | 2: Limited impairment | 13.2 (38809) | 14.4 (27628) | 13.7 (66437) |
|  | 3: Extensive assistance required (I) | 5.2 (15153) | 6.9 (13256) | 5.9 (28409) |
|  | 4: Extensive assistance required (II) | 5.0 (14594) | 5.1 (9805) | 5.0 (24399) |
|  | 5: Dependent | 3.6 (10441) | 3.6 (6829) | 3.6 (17270) |
|  | 6: Total Dependence | 0.8 (2498) | 0.9 (1807) | 0.9 (4305) |
| IADL difficulty scale | 0: No difficulty in any IADLs | 5.1 (14908) | 5.8 (11207) | 5.4 (26115) |
|  | 1: Some difficulty in one IADL | 7.7 (22591) | 5.4 (10328) | 6.8 (32919) |
|  | 2: Some difficulty in two IADLs | 14.3 (41888) | 11.7 (22484) | 13.3 (64372) |
|  | 3: Some difficulty in all three IADLs | 2.1 (6102) | 2.0 (3803) | 2.0 (9905) |
|  | 4: Great difficulty in one IADL | 20.7 (60673) | 17.2 (33024) | 19.3 (93697) |
|  | 5: Great difficulty in two IADLs | 37.0 (108645) | 40.4 (77526) | 38.4 (186171) |
|  | 6: Great difficulty in all three IADLs | 13.2 (38914) | 17.4 (33314) | 14.9 (72228) |
| Bladder/bowel incontinence | Incontinence present in last 7 days | 43.8 (128695) | 34.1 (65409) | 40.0 (194104) |
| Pain intensity | Mild or no pain | 49.3 (144917) | 59.1 (113302) | 53.2 (258219) |
|  | Moderate | 36.2 (106253) | 30.1 (57703) | 33.8 (163956) |
|  | Severe | 11.3 (33100) | 8.4 (16151) | 10.1 (49251) |
|  | Pain is horrible | 3.2 (9451) | 2.4 (4530) | 2.9 (13981) |
| **Cognitive abilities** | |  |  |  |
| Cognitive Performance Scale | 0: Intact | 44.4 (130370) | 41.0 (78515) | 43.0 (208885) |
|  | 1: Borderline intact | 16.4 (48145) | 16.4 (31435) | 16.4 (79580) |
|  | 2: Mild impairment | 16.2 (47686) | 16.9 (32374) | 16.5 (80060) |
|  | 3: Moderate impairment | 18.5 (54266) | 20.2 (38756) | 19.2 (93022) |
|  | 4: Moderately severe impairment | 1.4 (4107) | 2.0 (3914) | 1.7 (8021) |
|  | 5: Severe impairment | 2.5 (7300) | 2.8 (5447) | 2.6 (12747) |
|  | 6: Very severe impairment | 0.6 (1847) | 0.7 (1245) | 0.6 (3092) |
| Had delirium in the last 90 days | Yes | 5.8 (17005) | 7.3 (13919) | 6.4 (30924) |
| **Mood and behavior** | |  |  |  |
| Wandering behavior | Yes | 2.4 (7143) | 3.2 (6101) | 2.7 (13244) |
| Verbally and/or physically abusive | Yes | 2.8 (8180) | 4.6 (8738) | 3.5 (16918) |
| Socially inappropriate/disruptive | Yes | 1.5 (4400) | 2.1 (3992) | 1.7 (8392) |
| Depression rating scale (categorized) | Possible depression | 18.3 (53651) | 15.0 (28786) | 17.0 (82437) |
| **Comorbidities** | |  |  |  |
| Has Alzheimer's or dementia | Yes | 21.0 (61716) | 21.7 (41534) | 21.3 (103250) |
| Has any cardiovascular diseases^[[6]](#footnote-6)^ | Yes | 36.6 (107448) | 47.0 (90140) | 40.7 (197588) |
| Has Parkinson’s disease | Yes | 2.4 (6978) | 5.1 (9726) | 3.4 (16704) |
| Has any fractures | Yes | 15.3 (45009) | 8.4 (16059) | 12.6 (61068) |
| Has cancer (excluding skin cancer) | Yes | 14.5 (42625) | 21.9 (41900) | 17.4 (84525) |

1. All p-values for differences between women and men care-receivers are <0.0001 unless otherwise specified [↑](#footnote-ref-1)
2. IQR: inter-quartile range [↑](#footnote-ref-2)
3. Difference between women and men care-receivers are significant at p = 0.0078 [↑](#footnote-ref-3)
4. Difference between women and men care-receivers are significant at p = 0.0043 [↑](#footnote-ref-4)
5. All p-values for differences between women and men care-receivers are <0.0001 [↑](#footnote-ref-5)
6. Includes stroke, coronary heart diseases, congestive heart failure, or peripheral vascular disease [↑](#footnote-ref-6)
